# Factors influencing childhood immunisation uptake in Africa: a systematic review

**DOI:** 10.1186/s12889-021-11466-5

**Published:** 2021-07-28

**Authors:** Abubakar Nasiru Galadima, Nor Afiah Mohd Zulkefli, Salmiah Md Said, Norliza Ahmad

**Affiliations:** Department of Community Health, Faculty of Medicine and Health Sciences, University Putra Malaysia, Serdang, Malaysia

**Keywords:** Childhood immunisation, Uptake, Africa

## Abstract

**Background:**

Vaccine preventable diseases are still the most common cause of childhood mortality, with an estimated 3 million deaths every year, mainly in Africa and Asia. An estimate of 29% deaths among children aged 1–59 months were due to vaccine preventable diseases. Despite the benefits of childhood immunisation, routine vaccination coverage for all recommended Expanded Programme on Immunization vaccines has remained poor in some African countries, such as Nigeria (31%), Ethiopia (43%), Uganda (55%) and Ghana (57%). The aim of this study is to collate evidence on the factors that influence childhood immunisation uptake in Africa, as well as to provide evidence for future researchers in developing, implementing and evaluating intervention among African populations which will improve childhood immunisation uptake.

**Methods:**

We conducted a systematic review of articles on the factors influencing under-five childhood immunisation uptake in Africa. This was achieved by using various keywords and searching multiple databases (Medline, PubMed, CINAHL and Psychology & Behavioral Sciences Collection) dating back from inception to 2020.

**Results:**

Out of 18,708 recorded citations retrieved, 10,396 titles were filtered and 324 titles remained. These 324 abstracts were screened leading to 51 included studies. Statistically significant factors found to influence childhood immunisation uptake were classified into modifiable and non-modifiable factors and were further categorised into different groups based on relevance. The modifiable factors include obstetric factors, maternal knowledge, maternal attitude, self-efficacy and maternal outcome expectation, whereas non-modifiable factors were sociodemographic factors of parent and child, logistic and administration factors.

**Conclusion:**

Different factors were found to influence under-five childhood immunisation uptake among parents in Africa. Immunisation health education intervention among pregnant women, focusing on the significant findings from this systematic review, would hopefully improve childhood immunisation uptake in African countries with poor coverage rates.

**Supplementary Information:**

The online version contains supplementary material available at 10.1186/s12889-021-11466-5.

## Background

Vaccine Preventable Diseases (VPDs) are still the most common cause of childhood mortality with an estimated 3 million deaths every year, mainly in Africa and Asia [1]. A study conducted by the World Health Organization (WHO) and United Nations International Children’s Emergency Fund (UNICEF) in 2014, reported that an estimate of 29% deaths among children aged 1–59 months were due to vaccine preventable diseases [2]. In 2014, there were 24.1 million reported cases of pertussis, with the African region accounting for the highest proportion of 7.8 million (33%) cases [3].

Immunisation is considered to be one of the most successful and cost-effective public health sustainable interventions for human beings against diseases that affect our health [4]. Routine immunisation plays a key role to significantly reduce child mortality due to vaccine preventable diseases. WHO revealed that immunisation has been estimated to prevent 3 million deaths globally every year [5]. Between the years 2000 and 2016, a decrease of 84% in the measles mortality rate was recorded worldwide due to measles vaccination [6]. Likewise, a reduction in pertussis mortality was also recorded globally from 390,000 deaths in 1999 among children younger than 5 years of age to 160,700 deaths in 2014 as a result of vaccine effectiveness against pertussis [6, 7].

According to the Expanded Programme on Immunization (EPI), every child in Africa must receive one dose of Bacillus Calmette Guerin (BCG), Oral Polio Vaccine (OPV0) and Hepatitis B Vaccine (HBV1) at birth, Penta1 & OPV1 at 6 weeks of age, Penta2 & OPV2 at 10 weeks of age, Penta3 & OPV3 at 14 weeks of age and measles and yellow fever at 9 months of age. Despite the benefits of childhood immunisation, routine vaccination coverage for all recommended EPI vaccines has remained poor in some African countries such as Nigeria (31%; 2018), Ethiopia (43%; 2019), Uganda (55%; 2016) and Ghana (57%; 2014). The coverage is higher in some of the African countries, such as in Tanzania in the year 2016 and Kenya in 2014 (75 and 78%, respectively) [8–13]. Diphtheria Pertussis and Tetanus (DPT3) coverage is also low in African countries such as Nigeria (50%) [8]. However, these coverages are still below the targets endorsed by WHO in the 2012 Global Vaccine Action Plan, which aimed to ensure delivery of universal access to immunisation with associated targets reaching 90% of the national vaccination coverage and at least 80% vaccination coverage in every district [14].

Previous observational studies conducted among African countries and other parts of the world highlighted various factors that influenced childhood immunisation uptake. These factors are socio-demographic factors including maternal age, maternal educational status, paternal educational status, mother’s marital status, maternal occupation, family income, wealth index and ethnicity [15–18] and obstetric factors including antenatal care follow-up, postnatal care follow-up, preceding birth interval and place of delivery [18–21]. Despite the poor childhood immunisation uptake in African countries, no current systematic review has been conducted that focuses on describing, in detail, the factors influencing childhood immunisation uptake in Africa. Therefore, the aim of this study is to collate evidence on the factors that influence childhood immunisation uptake in Africa as well as to provide evidence for future researchers in developing, implementing and evaluating intervention among African populations, which will improve childhood immunisation uptake. This study will assist in developing health promotional programs and policies on childhood immunisation in Africa.

## Methods

This systematic review was conducted and reported in accordance with published Reporting Items for Systematic Reviews and Meta-analysis (PRISMA) [22].

### Electronic database search

We designed and implemented a comprehensive systematic literature search with the assistance of an experienced librarian using a well-developed strategy. The following databases were searched on the same date, from date of inception to 26th of October 2020: Medline, PubMed, CINAHL and Psychology & Behavioral Science Collection. The search strategy comprised a combination of medical subheading (MeSH) terms and keywords: childhood immunization uptake, factors, influencing or affecting, child or newborn or infant or baby, immunisation or vaccines or vaccination or pentavalent vaccine or Penta vaccine or Bacillus Calmette Guerin vaccine or BCG or Diphtheria Tetanus and Pertussis or DTP or Oral Polio vaccine or OPV or Measles vaccine or Yellow fever vaccine or Pneumococcal Conjugate vaccine or PCV or Hepatitis B vaccine or Hep B vaccine, uptake or adherence or compliance or coverage (Supplemental file 1).

The search strategy was developed in Medline and adapted for the other databases in order to account for differences in indexing. We restricted it to humans in the search process. Reference lists of included studies were also searched.

We included any observational (cross-sectional, case-control and cohort), mixed method study (convergent, exploratory and explanatory type) and qualitative study design (phenomenological study and case study) conducted in Africa published in the English language with findings relating to childhood immunisation uptake. The participants of these studies were caregivers with children under 5 years of age. The included studies also reported data on association between possible predictors and childhood immunisation or provided details of any non-compliance (vaccine refusal). We also included peer-reviewed full text publications reporting an association between at least one individual factor and uptake of childhood immunisation. Moreover, no restrictions were imposed on the year of publication.

We excluded articles without any description of study design, intervention studies (randomised controlled trial and quasi experimental design), review articles or systematic reviews and studies which made no mention of any of the Expanded Programme on Immunization target vaccines according to the National Program on Immunization (Table 1).

**Table 1:** Eligibility criteria

N	Inclusion criteria	Exclusion criteria
1.	Quantitative study designs	Articles without any description of study design
2.	Qualitative study designs	Intervention studies
3.	Studies in Africa	Review articles or systematic review
4.	Published in English language	Studies without any of the EPI target
5.	Caregivers	
6.	Children less than five years of age	
7.	Peer-reviewed full text publications	

### Description of study outcomes

Parental socio-demographic factors: maternal age, maternal education, paternal education, income, place of residence, maternal occupation and religion; child socio-demographic factors: child age and gender; religion and cultural beliefs; obstetric history: place of delivery, antenatal care follow-up and postnatal care follow-up; health care system: distance to hospital and availability of vaccine; knowledge; outcome expectation and self-efficacy (Table 2).

**Table 2:** Operational Definitions Table

No.	Variables	Definition
	Sociodemographic of respondent	This refers to the sociodemographic of respondent’s which include: maternal age, marital status, no children, maternal educational status, paternal educational status, maternal occupation, religion, ethnicity, monthly income and place of resident.
1.	Maternal age	This refers to the age of mother at the time of delivery which is been categorized into two different groups; below 20 or above 20 years of age (Negussie, Kassahun, Assegid & Hagan, 2015) [23].
2.	Marital status	This refers to the respondent’s official marital status which is been categorized into five different groups single, married, divorced or widowed (Anokye et al., 2018) [24].
3.	No of children	This refers to the numbers of children having per mother (Oliveira, Martinez & Rocha, 2014) [25].
4.	Maternal educational status	This refers to educational status of mother which could be no-formal education, primary school education, secondary school education or tertiary education (Kiptoo, Esilaba, Kobia & Ngure, 2015) [26].
5.	Paternal educational status	This refers to educational status of father which could be no-formal education, primary school education, secondary school education or tertiary education (Legesse & Dechasa, 2015) [16].
6.	Maternal occupation	This refers to occupational status of mothers and it is been classified into four categories; house wife, student, trader, civil servant or farmer (Legesse & Dechasa, 2015) [16].
7.	Religion	The respondent’s religion status for this study is categorized into Muslim and non-Muslim.
8.	Ethnicity	The respondent’s ethnicity for this study is categorized into Hausa and non-Hausa
9.	Monthly income	This refers to the amount of income a family earn in a month and in this study, it is categorized into less than 18,000 or above 18,000 naira (Nigerian currency).
10.	Place of resident	Area of residence was described as a place where people are living which could either be a rural or urban area (Kiptoo, 2015) [26].
	Obstetric history of respondent	This refers to the number of times attended ANC follow-up (Legesse & Dechasa, 2015) [16], birth interval between last born and current pregnancy (Rahman & Obaida-Nasrin, 2010) [21] and whether or not mothers receive any Tetanus toxoid vaccine (Adedire et al., 2016) [27]. In this study, number of times attended ANC follow-up is categorized into either less than three times or more-than three times. Birth interval between last born and current pregnancy was classified into three categories: first pregnancy, less than 48 months or more than 48 months. For whether or not mothers receive any Tetanus toxoid vaccine, it was classified as either yes or no.
	Health care system of respondent	This refers to health care infrastructure or resourcing which includes: distance to health facility (Legesse & Dechasa, 2015) [16], attitude of the hospital staff (Chambongo, Nguku, Wasswa & Semali, 2016) [28], accessibility of vaccine site (Animaw et al., 2014) and mode of transportation to reach hospital (Odutola et al., 2015) [29]. In this study, distance to health facility is classified into either less than 30 min or more than 30 min, attitude of hospital staff could either be good or poor, hospital accessibility either yes or no and means of transportation to hospital either car/motorcycle or walking.
	Maternal knowledge	This refers to the knowledge of mother’s regarding general knowledge on childhood immunization, knowledge on child immunization schedule, knowledge on vaccine side effects and management and knowledge on sign, symptoms and mode of transmission of VPDs.
	Maternal attitudes	This refers to the attitudes of mother’s towards childhood immunization uptake.
	Maternal outcome expectation Maternal self-efficacy Environmental factors	This refers to the outcome expectation of mother’s towards childhood immunization uptake (important of vaccinating a child and consequences that may arise for non-compliance). This refers to the self-efficacy of mother’s towards childhood immunization uptake. This refers to factors other than cognitive including cultural beliefs, religious beliefs and health care system.
	Cultural beliefs	This refers to the cultural beliefs of respondent’s towards childhood immunization uptake.
	Religious beliefs	This refers to the beliefs on religious regulations of respondent’s towards childhood immunization uptake.

### Selection of studies

All searched articles were evaluated for eligibility in order to be included in the review. The main researcher first removed all duplicates and screened the titles using Microsoft Excel. Abstract screening was then conducted independently by two reviewers. The full text of any article considered potentially relevant was also retrieved independently by the two reviewers. Consensus was reached by discussion or involvement of a third reviewer when there were differences of opinion.

### Data extraction

A data extraction form was designed and piloted for this review. The form was used to extract the following data: study characteristics such as authors’ name and year of publication, study location, method, study design, sample size, results and factors. Data was extracted independently by the two reviewers to ensure accuracy. Any difference of opinion was resolved by discussion or involvement of a third reviewer.

### Categorisation of factors associated with immunisation coverage

The qualitative synthesis of the factors influencing childhood immunisation uptake among parents of children under-five years of age are categorised into two groups, namely modifiable and non-modifiable factors. Modifiable factors are considered to be factors that are intrinsic to mothers/caregivers (can be changed through health education intervention targeting mothers/caregivers) while non-modifiable factors are considered to be factors that are extrinsic to mothers/caregivers (cannot be changed through health education intervention targeting mothers/caregivers). This classification would enable researchers to plan an appropriate health education intervention, with the use of appropriate health behavioural theory.

### Assessment of methodological quality

ANG and NZ assessed the methodological quality for each of the included studies using the criteria outlined in the Joanna Briggs Institute (JBI) for both quantitative and qualitative studies [30], which includes 8 items for cross-sectional study design and 10 items for both case-control and qualitative study designs respectively*.* Any disagreement was resolved by discussion. In this review, we grouped articles into three categories: high, moderate and low quality for their methodological qualities using the JBI critical appraisal tools; studies scoring > 70% were considered as high quality, 60–69% were moderate quality and < 60% were low quality. This review may be prone to publication bias as grey literature/unpublished literature and studies that are not published in the English language were not considered.

### Data synthesis

No meta-analysis was conducted, however, the results obtained from the systematic review were synthesised and placed in a logic framework. The various results obtained from the systematic review were shown in the logic framework and discussed in a narrative synthesis manner. The descriptive information and data for each of the included studies were captured using a table formulated by the researchers. These included study author, year and location; method; study design; sample size; results and factors. ANG synthesis ed the data and created a table with input from both NZ and SS. ANG and NZ reported factors under two different categories namely: modifiable and non-modifiable factors.

In this review, a wide range of AOR and 95% confidence intervals (upper limit and lower limit) from different studies were reported and confidence interval that does not include 1 and *p*-value less than 0.05 with AOR greater than 1 are considered as statistically significant.

## Results

**Fig 1:**
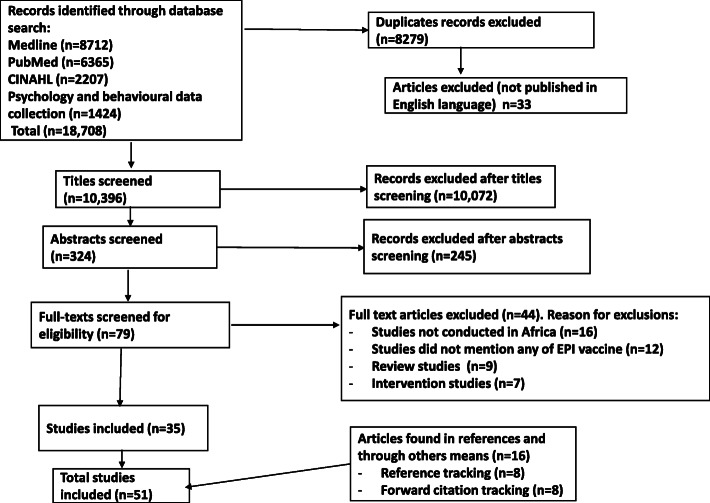
PRISMA flow diagram for the identification, screening, eligibility, and inclusion of studies

Of 18,708 recorded citations retrieved, 8279 duplicates and 33 articles published in languages other than the English language were removed. After screening 10,396 titles, 324 records remained and their abstracts were screened. Seventy-nine records remained after abstracts were screened and their full texts were assessed for eligibility. We excluded 44 studies in total, of which 16 studies were not conducted in Africa, 12 studies did not make mention of the EPI vaccine, 9 were review studies and 7 were intervention studies. In total, 51 studies were included, of which 35 were from the main database search, 8 from forward citation tracking and 8 from reference tracking. All the included studies looked into factors influencing childhood immunisation uptake in one of the African countries (Fig. 1). Table 3 contained a summary of the factors that influence under-five childhood immunisation uptake among mothers/caregivers. The studies used different study designs to investigate the factors that influence under-five childhood immunisation uptake among parents.

**Table 3:** Summary of systematic review of factors influencing childhood immunization uptake in Africa

Authors/year/Location	Method	Study design	Sample size	Results	Factors	Study quality
Sanou, 2009 (Nouna District, Burkina Faso) [31]	Nouna Health Research Centres Demographic Surveillance System (DSS).	Cross-sectional	*n* = 476	Knowledge of the preventive objectives of immunization: Illiterate parent vs literate parents 7.8% Vs 90%, *p* = 0.030 completely immunized; Availability of a vaccination record document: Yes vs no: OR = 2.381; 95% CI: 1.436–3.948, *p* = 0.001; Religion: Muslims Vs Others OR = 1.813; 95% CI: 1.102–2.985, p = 0.019; Marital status: Monogamous parent’s vs polygamous parents; 61.4% vs 34.2%, *p* < 0.05; Economic status: 4th quartile vs 1st, 2nd and 3rd: OR = 2.100; 95% CI: 1.242–3.554, p = 0.006	1. Knowledge 2. Availability of child immunization record 3. Religion 4. Marital status 5. Economic status	High
Chiabi, 2017 (Yaounde, Cameroon) [32]	Pre-tested questionnaire and children’s vaccination booklets	Cross-sectional	*n* = 400	Maternal educational status: Higher education vs primary and secondary (AOR = 7.0; 95% CI: 2.16–22.68, *p* = 0.001). Paternal occupational status: Employed vs unemployed (AOR = 12.39; 95% CI: 2.21–69.26, *p* = 0.004)	1. Maternal education 2. Paternal occupation	High
Mwamba, 2017 (Kinshasa, Democratic Republic of Congo) [33]	Structured interview	Cross-sectional	*n* = 1224	Distance to health facility: < 30 min vs > 30 min *p* = 0.04; Mothers thought child vaccine is up-to date: Yes vs No < 0.001.	1. Distance to health facility 2. Mothers thought	High
Aregawi, 2017 (Laelay Adiabo District, Northern Ethiopia) [34]	Structured questionnaire	Case-control	*n* = 270	Maternal knowledge on Immunization: Good vs poor (AOR = 3.3,95% CI:1.87–7.43). Post-natal care follow-up: Yes vs no (AOR = 5.2,95%CI:2.36–11.46). participation in women’s developmental groups: Good vs poor (AOR = 3.3,95%CI 1.54–7.08). Health extension worker visit: Yes vs no (AOR = 2.68,95%CI:1.30–5.51). Distance to health facility: < 30 min vs > 30 min (AOR =3.56,95%CI:1.58–8.01). Other reasons for defaulting: Child illness 21.1%; Forgetfulness 17.80%; Inconvenience time 8.9%; Lack awareness about vaccine 7.80%; Not knowing return for 2nd and 3rd dose 7.80%; Vaccine not available 5.60% and Fear of side effect 4.40%.	1. Maternal knowledge on immunization 2. Post-natal care 3. Participation in women’s developmental groups 4. Health extension worker visit 5. Attitude 6. Lack of awareness 7. Lack of knowledge on EPI schedule 8. Availability of vaccine	High
Animaw, 2014 (Arba Minch town and Zuria District, Southern Ethiopia) [29]	Semi structured questionnaire and child immunization record	Cross-sectional	*n* = 630	Maternal educational status: Primary or above vs no education (AOR = 2.22; 95% CI 1.31,3.76). Accessible vaccination site: Yes vs no (AOR = 4.54; 95%CI:2.34,8.77). Nearest vaccine site: Health post vs outreach (AOR = 1.89; 95% CI: 1.07,3.33).	1. Maternal education 2. Accessible vaccine site 3. Nearest vaccine site	High
Etana, 2012 (Ambo Woreda, Central Ethiopia) [35]	Structured questionnaire and child immunization record	Cross-sectional	*n* = 536	Maternal Knowledge on benefit of immunization: Yes vs no (OR = 4.5; 95% CI: 2.5,7.9). Maternal Knowledge on age to start immunization: Yes vs no (AOR = 2.9, 95% CI: 1.9,4.6). Maternal knowledge on age to complete child immunization: Yes vs no (AOR = 4.3; 95%CI:2.3,8.0). ANC follow-up: Yes vs no (AOR = 2.4; 95% CI:1.2,4.9). Maternal knowledge on total immunization session: Yes vs no (OR = 1.7; 95%CI:1.1,2.5). Place of delivery: Hospital vs home (AOR = 2.1; 95% CI: 1.3,3.4).	1. Maternal knowledge on benefit of vaccine 2. Knowledge on age to start child immunization 3. Knowledge on age to complete child immunization 4. ANC follow-up 5. Maternal knowledge on total immunization session 6. Place of delivery	High
Negussie, 2016 (Arbegona district, southern Ethiopia) [23]	Structured questionnaire and focused group discussion	Mixed method	*n* = 548	**Case-control findings** Maternal Age: > 19 years vs < 19 years (AOR = 9.54; 95% CI: 5.03, 18.09, *p* = 0.001). Knew the benefits of immunization: Yes vs no (AOR = 5.51; 95% CI:1.52, 19.94. *p* = 0.009). Perception about vaccine side effects: Positive vs negative (AOR = 1.92; 95% CI:1.01, 3.70). Birth order: 1 vs 2–4 (AOR = 3.64; 95% CI:1.63, 8.14, *p* < 0.001); 1 vs > 5 (AOR = 5.27; 95% CI:2.20, 12.64, *p* = 0.002). **Qualitative findings** Maternal migration, fear of vaccine side effect, unavailability of vaccine, knowledge on EPI schedule.	1. Maternal age 2. Knowledge on benefit of immunization 3. Perception about vaccine side effect 4. Birth order 5. Maternal migration 6. Availability of vaccine 7. knowledge on EPI	High
Lakew, 2015 (Ethiopia) [20]	Ethiopian Demographic and Health Survey (EDHS) women questionnaire	Cross-sectional	*n* = 1927	Source of immunization information: Immunization record vs mothers self-report (AOR = 7.7; 95% CI: 5.95–10.06). postnatal check-up: Yes vs no (AOR = 1.8; 95% CI: 1.28–2.56). women’s awareness of community conversation program: Yes vs no (AOR = 1.9; 95% CI: 1.44–2.49). wealth index: Rich vs poor (AOR = 1.4, 95% CI:1.06–1.94).	1. Source of information 2. PNC check-up 3. Awareness 4. Wealth index	High
Mohamud, 2014 (Jigjiga District, Somali National Regional State, Ethiopia) [36]	Structured questionnaire	Cross-sectional	*n* = 582	Maternal age: > 20 years vs < 19 years (AOR = 2.19; 95% CI:1.26,3.83). Maternal literacy: Literate vs illiterate (AOR = 3.06; 95% CI:1.64,5.71). Place of residence: Urban vs rural (AOR = 2.04; 95% CI:1.33,3.13). Maternal TT vaccine: Yes vs no (AOR = 2.43; 95% CI: 1.56,3.77). Place of delivery: Hospital vs home (AOR = 2.02; 95% CI:1.24,3.28). Household visit by health workers: Yes vs no (AOR = 1.92; 95% 1.17,3.16).	1. Maternal age 2. Maternal literacy 3. Place of residence 4. Maternal TT vaccine 5. Place of delivery 6. House hold visit by health workers	High
Abebe, 2019 (Bassona Worena Woreda, Amhara Region, Ethiopia) [37]	Structured questionnaire and focused group discussion	Mixed method	*n* = 575	**Cross-sectional findings** Maternal age: > 40 years versus < 40 years (AOR = 1.9; 95% CI 1.12, 5.83). Awareness about immunization: Yes vs no (AOR = 2.8; 95% CI 1.67, 9.34). ANC follow-up: Yes vs no (AOR = 3.67; 95% CI 1.96, 6.78). Availability of health facility: Yes vs no (AOR = 1.49; 95% CI 1.06, 8.12). **Qualitative findings** Lack of awareness on immunization; no faith on immunization; ANC follow-up; availability of health facility	1. Maternal age 2. Awareness 3. ANC follow-up 4. Availability of health facility 5. Lack of faith	High
Zewdie et al., 2016 (Hadiya zone, Southern Ethiopia) [38]	Phenomenological method	Qualitative	*n* = 14	Lack of awareness on immunization; no faith on immunization; ANC follow-up; availability of health facility; knowledge of benefits of immunization; knowledge of vaccination schedules and service arrangements; lack of social support from family; loss of vaccination card; problems with vaccine supply and service arrangement; health systems and health care provider factors; poor counselling and client-provider relationships are influencing childhood immunization uptake.		High
Payne, 2013 (Gambia) [39]	Farafenni Health and Demographic Surveillance System (FHDSS).	Cross-sectional	*n* = 7363	Ethnic group: Wolof vs Mandika (AOR = 1.52; 95% CI:1.28–1.81, *p* < 0.001). Wealth index: Quintile 5 vs Quintile 1 (AOR = 1.49; 95% CI: 1.09–2.04, *p* = 0.011).	1. Ethnic group 2. Wealth index	Moderate
Odutola, 2015 (Western region of Gambia) [40]	Structured questionnaire	Cross-sectional	*n* = 1154	Place of birth: Hospital vs home (AOR = 1.47; 95% CI: 1.05–2.07, *p* = 0.001). Mode of transportation: Public transport vs walking (AOR = 1.54; 95% CI:1.20–1.97, *p* = 0.02). Birth order: > 2 vs < 2 (AOR:1.37; 95% CI: 1.04–1.79).	1. Place of birth 2. Mode of transportation 3. Birth order	High
Bosu, 1997 (Komenda-Edina-Eguafo-Abrem District of Ghana) [41]	Structured questionnaire and focused group discussion	Mixed method	*n* = 469	**Cross-sectional findings** Knowledge of EPI diseases: Inadequate Vs adequate: 30.35% vs 17.58% children not fully immunized. Mothers who never attend child immunization (*n* = 74): financial difficulties (37.8%); baby too young (14.9%); mothers travelled out of community (13.5%); mothers too busy (4.1%)	1. Knowledge on EPI diseases 2. Financial difficulties 3. Mothers attitude	Moderate
Anokye, 2018 (Koforidua, Ghana) [24]	Structured questionnaire	Cross-sectional	*n* = 280	Marital status: Married vs divorced (AOR = 3.01; 95% CI: 1.59–58.2, *p* = 0.048); Employment status: Working part-time vs unemployed (AOR = 2.28; 95% CI:1.031–9.11, *p* = 0.049); Maternal income: > 100 cedes vs < 100 cedes (AOR = 2.41; 95% CI:1.56–2.01).	1. Marital status 2. Employment status 3. Income	High
Mutua, 2011 (Korogocho and Viwandani slums of Nairobi, Kenya) [42]	Nairobi Urban Health and Demographic Surveillance System (NUHDSS)	Cross-sectional	*n* = 1848	Place of delivery: Hospital vs home (OR = 1.27; 95% CI: 1.002,1.619). Maternal education: Complete primary school vs not complete (OR = 1.3024; 95% CI: 1.011,1.676). Maternal age: > 20 years vs < 20 years (OR = 1.48; 95% CI: 1.057,2.079).	1. Place of delivery 2. Maternal education 3. Maternal age	High
Pertet, 2018 (pastoralist community of Kenya) [43]	An interviewer-administered questionnaire	Cross-sectional	*n* = 515	lack of vaccines, *p* = 0.002; location of health facility, p = <.001; nomadic lifestyle: OR = 9.0; 95% CI: 1.11, 72.66, *p* = 0.006	1. Availability of vaccine 2. Location of health facility 3. Nomadic lifestyle	High
Jani, 2008 (Southern Mozambique) [44]	Face to face Interview	Cross-sectional	*n* = 668	Distance to health facility: Near vs far away (OR = 3.64; 95% CI: 1.71,7.74, *p* = 0.001). Maternal schooling: Yes vs no (OR = 2.24; 95% CI: 1.41,3.56, p = 0.001). Knowledge on EPI: Yes vs no (OR = 2.02; 95% CI: 1.19,3.42, p = 0.009). Religious beliefs: Yes vs no (OR = 1.65; 95% CI: 1.15,2.36, *p* = 0.004). Child born: Inside Mozambique vs outside (OR = 5.20; 95% CI: 3.35,11.51, *p* < 0.001). Place of delivery: Hospital vs home (OR = 1.78; 95% CI: 1.28,3.36, *p* = 0.03). Marital status: Married vs divorced or widowed (OR = 1.68; 95% CI: 1.07,2.64, *p* = 0.02).	1. Distance to HF 2. Maternal schooling 3. Knowledge on EPI 4. Religious beliefs 5. Child born location 6. Place of delivery 7. Marital status	High
Umeh, 2018 Northern Nigeria [45]	Face to face interview	Cross-sectional	*n* = 396	**Compliant vs non-compliant** Satisfaction with immunization p = 0.001; refusal to vaccination p = 0.001; doubt about Immunization p = 0.001; worries about Vaccination Safety p = 0.001; knowledge on importance of vaccination p = 0.001; seriousness of VPDs *p* = 0.045.	1. Immunization satisfaction 2. Attitudes 3. Knowledge on important of vaccination 4. Seriousness of VPDs	High
Oladokun, 2010 (Ibadan, Nigeria) [46]	Face to face interview	Cross-sectional	*n* = 248	Maternal education: Primary vs none: OR = 5.90; 95% CI: 1.87,17.92, *p* = 0.002. Religion: Christianity vs Islam: OR = 3.05; 95%: 1.20,7.81, *p* = 0.019. Gender of child: Male vs female: OR = 2.98; 95% CI: 1.21, 7.35, *p* = 0.017. Mothers Beliefs and attitudes on Immunization: 248 defaulters: availability of vaccines 65 (26.2%); lack of knowledge on EPI 41 (16.5%); inconvenient time 34 (13.7%); lack of knowledge on benefit of immunization 24 (9.7%); child ill 25 (10.1%); Immunization is waste of time 129 (52%); immunization is harmful to children 81 (32.7%)	1. Maternal education 2. Religion 3. Child gender 4. Mothers beliefs and attitude	Moderate
Babalola, 2008 South and Northern Nigeria [47]	Face to face interview	Cross-sectional	*n* = 1472	Place of delivery: Hospital vs home (OR = 2.54). child immunization record: Yes vs no (OR = 2.10). Immunization ideation: High vs low (OR = 6.04).	1. Place of delivery 2. Child immunization record 3. Immunization ideation	Moderate
Odusanya, 2008 Edo State, Nigeria [48]	Interviewer administer questionnaire	Cross-sectional	*n* = 339	Child immunization record: Yes vs no (p = 0.002)	1. Child immunization record	High
Taiwo, 2017 Kaduna State, Nigeria [49]	Semi-structured interviewer-administered questionnaire	Cross-sectional	*n* = 379	Maternal education: educated vs uneducated (AOR = 1.90; 95% CI: 1.11,3.28). Maternal perception on immunization: Good vs poor (AOR = 2.60; 95% CI: 1.50,4.51). Maternal knowledge on immunization: Satisfactory vs unsatisfactory (135 (35.6%) vs 244 (64.4%)).	1. Maternal education 2. Maternal perception 3. Maternal knowledge on immunization	Moderate
Oku, 2017 Northern and Southern Nigeria [50]	Case study model	Qualitative	*n* = 15	health workers shortages; training deficiencies; poor attitudes of health workers; long waiting times; attitudes among community members; engagement of traditional and religious institutions		High
Adedire, 2016 Ogun State, Nigeria [27]	Semi-structured questionnaire	Cross-sectional	*n* = 750	ANC follow-up: Yes vs no (AOR = 3.3; 95% CI:1.2, 8.3, p = 0.03). Maternal tetanus toxoid: At least a dose vs none (AOR = 3.2; 95% CI: 1.1,10.0, p = 0.04). Maternal knowledge on RI: Good vs poor (AOR = 2.4; 95% CI: 1.6,3.8, *p* < 0.001). Access to immunisation information in last 12 months: Yes vs no (AOR = 2.5, 95% CI:1.1, 2.5, *p* = 0.02).	1. ANC follow-up 2. Maternal TT 3. Maternal knowledge on RI 4. Accessibility	High
Adedokun, 2017 Nigeria [18]	Secondary analyses from the 2013 Nigeria Demographic and Health Survey (NDHS)	Cross-sectional	*n* = 5754	Maternal education: secondary or higher vs none (AOR = 2.14; 95% CI:1.59,2.86 secondary or higher vs primary: AOR = 1.42; 95% CI:1.14,1.76). Birth order 1st -3rd order vs 4th -6th order (AOR = 1.53; 95% CI:1.24,1.86). Access to health facility: Not a problem vs problem (AOR: 1.28;1.02,1.57).	1. Maternal education 2. Birth order 3. Accessibility	High
Antai, 2009 Nigeria [51]	Secondary analyses from the 2003 Nigeria Demographic and Health Survey (DHS)	Cross-sectional	*n* = 3725	Ethnicity: Hausa/Fulani vs Igbo (AOR = 2.47; 95% CI:1.28,4.76).	1. Ethnicity	High
Ijarotimi, 2018 Oyo State, Nigeria [52]	interviewer administered questionnaires	Cross-sectional	*n* = 449	Maternal educational status: > Primary vs none (AOR = 6.4; 95% CI:2.9,14.0). Maternal religion: Christian vs Muslims (AOR = 2.2; 95% CI: 1.3–3.7). Wealth index: Richest vs poorest (AOR = 14.5; 95% CI:8.2–20.5).	1. Maternal education 2. Religion 3. Wealth index	High
Chambongo, 2016 Ileje District, Tanzania [28]	Structured questionnaire	Cross-sectional	*n* = 380	Place of birth: Health facility vs home (AOR = 14.4; 95% CI:8.04–25.8). Perceived quality of vaccine provider client’s relationship: Positive vs negative (AOR = 1.86; 95% CI: 1.03–3.5). Satisfaction with vaccine services: Satisfied vs unsatisfied (AOR = 2.63; 95% CI:1.1–6.3).	1. Place of birth 2. Perceived quality of vaccine provider client’s relationship 3. Satisfaction with vaccine services:	High
Semali, 2010 Tanzania [53]	Secondary analyses from the 1990, 1996 and 2004 Tanzania DHS	Cross-sectional	*n* = 4471	Residence: Urban vs rural (AOR = 1.4; 95% CI: 1.0–1.9). Number of children under five years: < 2 vs > 2 (AOR = 1.4; 95% CI: 1.0–1.8). Wealth index: Least poor vs most poor (AOR = 1.9; 95% CI: 1.1–3.7).	1. Residence 2. Number of children under five years 3. Wealth index	Moderate
Vonasek, 2016 Rural Sheema District Southwest Uganda [54]	Face-to-face interviews	Cross-sectional	*n* = 476	Stated reasons to immunize children protect children from disease: Yes vs no (PR = 1.35; 95% CI: 1.01, 1.80).	1. Knowledge	Moderate
Kiptoo, 2015 East Pokot, Baringo, Kenya [26]	Structured questionnaire	Cross-sectional	*n* = 298	Maternal level of education: primary vs none (OR = 3.55; 95% CI: 1.49–8.47; *p* = 0.0049). knowledge on immunization schedule: yes vs no (OR = 9.04; 95% CI: 1.37–7.87; *p* < 0.0001). Nomadic lifestyle: yes vs no (OR = 11.06; 95% CI: 4.29–28.54; *p* < 0.0001). Distance to health facility: < 1-h vs > 1 h (OR = 18.24; 95% CI: 5.56–59.80; *p* < 0.0001). Area of residence: urban vs rural (OR = 12.3; 95% CI: 4.77–31.73; *p* < 0.0001). Place of birth: hospital vs home (OR = 4.5; 95% CI: 1.7–11.61; *p* < 0.0001).	1. Maternal level of education 2. Knowledge on immunization schedule 3. Nomadic lifestyle 4. Distance to health facility 5. Area of residence 6. Place of birth	High
Kagone, 2017 Nouna, North West Burkina Faso [55]	Nouna Health and Demographic Surveillance System (NHDSS)	Cross-sectional	*n* = 6579	Maternal educational status: educated vs non educated (AOR = 1.08; 95% CI: 1.02–1.13; p = 0.02)	1. Educational status	High
Gidado, 2014 Zamfara state, Nigeria [15]	Structured interviewer-administered questionnaire	Cross-sectional	*n* = 450	Satisfactory knowledge on routine immunization: yes vs no (AOR = 18.4; 95% CI = 3.6–94.7). Level of education: secondary education vs none (AOR = 3.6; 95% CI = 1.2–10.6)	1. Satisfactory knowledge on routine immunization 2. Level of education	Moderate
Duru, 2016 Imo state, Nigeria [56]	Semi structured, interviewer administered questionnaire	Cross-sectional	*n* = 743	Maternal age (year): 25–29 vs < 25 (OR = 2.1; 95% CI: 1.12–4.05; *p* < 0.01). Maternal level of education: primary vs none (OR = 7.5; 95% CI: 1.27–44.08, *p* < 0.05). Knowledge about immunization: good vs poor (OR = 37.71; 95% CI: 4.74–299.62; *p* < 0.0001).	1. Maternal age 2. Maternal level of education 3. Knowledge about immunization	Moderate
Legesse, 2015 Southeast Ethiopia [16]	Pre-tested, interviewer administered questionnaire	Cross-sectional	*n* = 591	Antenatal care follow-up: yes vs no (AOR = 3.7; 95% CI: 2.3–5.9). Maternal occupation: farmer vs housewife (AOR = 1.9; 95% CI: 1.1–3.1). Paternal level of education: > secondary vs illiterate (AOR = 3.1; 95% CI: 1.3–7.4). Family income: > 1000 52 USD vs < 52 USD (AOR = 3.2; 95% CI: 1.4–7.4). Distance to health facilities: < an hour vs > an hour (AOR = 3.1; 95% CI: 1.5–6.3). Ever discussed about immunization with HEWs: yes vs no (AOR = 2.4, 95% CI: 1.3–4.2). Maternal knowledge on immunization: good vs poor (AOR = 2.5; 95% CI: 1.5–4.2).	1. Antenatal care follow-up 2. Maternal occupation 3. Paternal level of education 4. Family income 5. Distance to heath facilities 6. Ever discussed about immunization with HEWs 7. Maternal knowledge on immunization	High
Oliveira, 2014 Angola [25]	interviewer administered questionnaire	Cross-sectional	*n* = 1209	Child age (years): > 1 vs < 1 (APR = 1.78; 95% CI: 1.53–2.07). Family size: 2–3 vs > 6 (APR = 1.34; 95% CI: 1.05–1.71). Knowledge of immunization programs: yes vs no (APR = 1.32; 95% CI: 1.07–1.63). Appliances: radio vs television or none (APR = 1.45; 95% CI: 1.05–1.99).	1. Child age 2. Family size 3. Knowledge of immunization programs 4. Appliances	High
Bbale, 2013 Uganda [19]	Uganda Demographic Health Survey (UDHS)	Cross-sectional	*n* = 7591	Maternal educational status: primary education vs no education (increase probability of fully immunized child 8–14%; *p* < 0.05); secondary education vs no education (increase probability of child receiving three doses of DPT and oral polio vaccines: 6–7%; *p* < 0.05); primary education vs no education (increase probability of child receiving three doses of oral polio vaccines: 7–11%; *p* < 0.01). Wealth index: rich vs poor (increase probability of child being vaccinated against polio and measles by 7%; *p* < 0.05).	1. Maternal educational status 2. Wealth index	Moderate
Gunnala, 2016 Nigeria [57]	Pre-tested, interviewer administered questionnaire	Cross-sectional	*n* = 7815	Common reported reason for non-vaccination: lack of maternal knowledge on vaccines and vaccination services (50%), poor maternal attitude towards immunization (16%), lack of access to routine immunization services (15%) and fear of side effects (9%).	1. Lack of maternal knowledge on vaccines 2. Poor maternal attitude towards immunization 3. Lack of access 4. Fear of side effects	High
Chris-Otubor 2016 Nigeria [58]	semi-structured questionnaire	Cross-sectional	*n* = 232	Maternal education: primary or secondary vs none; marital status: married vs single or separated or divorced; religion: Islam vs Christian, geopolitical zone: and the mother or the father of the child been immunized as children significantly influenced maternal knowledge on childhood immunization (*p* < 0.05).	1. Maternal education 2. Marital status 3. Religion 4. Geopolitical zone 5. Mother or father being immunized as children	High
Tadesse, 2009 Ethiopia [59]	structured questionnaire	Case-control	*n* = 264	Current postnatal care visit: yes vs no (AOR = 19.52; 95% CI: 1.68–226.29. Perceived health institution support: positive attitude vs negative attitude (AOR = 2.71; 95% CI 1.39–5.26). knowledge of immunization schedule: yes vs no (AOR = 3.01; 95% CI: 1.42–6.35). knowledge on OPV schedule: yes vs no (AOR = 6.52; 95% CI: 1.35–31.39). knowledge on measles: yes vs no (AOR = 34.72; 95% CI: 12.74–94.64). knowledge on benefit of vaccines: yes vs no (AOR = 6.36; 95% CI: 1.24–9.53).	1. Postnatal care visit 2. Perceived health institution support 3. Knowledge on immunization schedule 4. Knowledge on OPV schedule 5. Knowledge on measles 6. Knowledge on benefit of vaccines	High
Kio, 2016 Ogun state, Nigeria [60]	Structured pre-tested questionnaire	Cross-sectional	n = 120	Reason for defaulting: 52% or respondents are lacking knowledge on child immunization schedule, 47.5% reported lack of awareness on immunization in their areas, 54.2 reported negative cultural belief on immunization in their areas, 43.8% believes immunization to has adverse effects, 54.2% reported communicable diseases has to do nothing with routine immunization and 51% reported their children to be available for immunization only if the schedule is convenient for them	1. Lack of maternal knowledge on child immunization schedule 2. Lack of awareness on immunization 3. Cultural beliefs 4. Adverse effects 5. Lack of knowledge routine immunization 6. Convenient time	Moderate
Awosan, 2018 Sokoto state, Nigeria [61]	standardized, structured, interviewer- administered questionnaire	Cross-sectional	*n* = 220	55.5% of the respondents are having poor knowledge of the child that requires immunization and its benefits. 50.9% of the respondents are having poor knowledge on vaccine preventable diseases (VPDs). Knowledge on VPDs: good vs poor (85.2% vs 46.4% *p* < 0.05 children fully immunized).	1. Knowledge on immunization 2. Knowledge on VPDs	High
Ekure, 2013 Southwest, Nigeria [62]	interviewer-administered questionnaire	Cross-sectional	*n* = 36	> 30% of the respondents reported not to take their children back to complete RI if they develop any adverse effect and > 40% of the respondents reported not to allow their children to receive polio vaccine.	1. Fear of adverse effects 2. Poor attitude	High
Canavan, 2014 Uganda [63]	Uganda demographic Health Survey (UDHS)	Cross-sectional	*n* = 474	Maternal educational status: secondary school or higher vs no formal education (AOR = 3.39; 95% CI:1.20–9.51). Place of delivery: public hospital vs home (AOR = 3.94; 95% CI: 2.12–7.33).	1. Maternal educational status 2. Place of delivery	High
Omotora, 2012 Borno state, Nigeria [64]	Phenomenological method	Qualitative	n = 120	The main reasons for not fully supporting immunization program in some areas includes: inadequate adequate information about logistics and time of immunization programme, lack of adequate involvement of traditional and religious leaders and poor attitude of health workers. Mothers need incentives in order for them to take their children for immunization in forms of soap and complimentary health care services.	1. Inadequate adequate information about logistics and time of immunization programme 2. Lack of adequate involvement of traditional and religious leaders 3. Poor attitude of health workers. 4. Incentives	High
Tamirat, 2019 Ethiopia [65].	Ethiopia Demographic and Health Survey (EDHS, 2016).	Cross-sectional	*n* = 1909	Maternal employment status: employed vs unemployed (AOR = 1.62, 95% CI: 1.31, 2.0). Wealth index: rich vs poor (AOR = 1.44, 95% CI: 1.07, 1.94). Maternal education: Primary school vs no formal education (AOR = 1.38,95% CI: 1.07, 1.78). ANC follow-ups (AOR = 2.79, 95% CI:2.17 3.59). Place of delivery: hospital vs home (AOR = 1.76, 95% CI: 1.36, 2.24).	1. Maternal employment status 2. Wealth index 3. Maternal education 4. ANC follow-ups 5. Place of delivery	High
Kinfe, 2019 Ethiopia [66].	Ethiopia Demographic and Health Survey (EDHS, 2016).	Cross-sectional	*n* = 1929	Maternal education: Primary vs no formal education (AOR = 1.58, 95% CI: 1.1, 2.28). Paternal employment status: Employed vs unemployed (AOR = 2.1, 95% CI: 1.32, 3.35). ANC visit: yes vs no (AOR = 1.94, 95% CI: 1.31, 2.86). Availability of child immunization record: yes vs no (AOR = 1.61, 95% CI: 1.21, 2.16).	1. Maternal education 2. Paternal employment status 3. ANC visit 4. Availability of child immunization record	High
Girman, 2019 Ethiopia [67].	pretested interviewer-administered questionnaire	Cross-sectional	*n* = 620	Antenatal care visit: yes vs no (AOR = 2.75, 95%CI: 1.52–5.0). Maternal education: higher education vs no formal education (AOR = 2.39, 95%CI: 1.06–5.36). Maternal knowledge on immunization: Good vs poor (AOR = 3.70, 95%CI: 2.37–5.79). Distance to health facility: short vs long (AOR = 2.65, 95%CI: 1.61–4.36). Place of delivery: hospital vs home (AOR = 2.58, 95%CI: 1.66–3.99).	1. ANC visit 2. Maternal education 3. Maternal knowledge on immunization 4. Distance to health facility 5. Place of delivery	High
Akwataghibe, 2019 Nigeria [68].	Semi-structured interviews and focused group discussion	mixed methods	*n* = 215	Health service factors like absence of delivery services, shortage of health workers, unavailability of vaccines at scheduled times, and indirect costs of immunization contributed to low utilization.	1. Absence of delivery services 2. Shortage of health workers 3. Unavailability of vaccines at scheduled times 4. Indirect costs of immunization	High
Mekonnen, 2020 Ethiopia [69].	Interviewer-administered questionnaire	Cross sectional study design	*n* = 821	Maternal education: >secondary education vs no formal education (AOR = 2.391; 95% CI: 1.317–4.343). Wealth index: riche vs poor (AOR = 2.381; 95% CI: 1.502–3.773). ANC follow-up: yes vs no (AOR = 2.844; 95% CI: 1.310–6.174).	1. Maternal education 2. Wealth index 3. ANC follow-up	High

The majority of our epidemiological studies achieved at least 70% scores which is considered high [16, 18, 20, 23–29, 31–38, 40, 42–45, 48, 50–52, 55, 57–59, 61–69] and the overall risk of bias was moderate in 10 studies (Table 3) [15, 19, 39, 46, 47, 49, 53, 54, 56, 60]. Out of the 10 studies, 6 had a moderate risk of bias in the confounding domain and 4 had a moderate risk of bias in the outcome assessment domain. However, none of the included epidemiological studies demonstrated either a serious or low risk of bias < 60%.

### Study locations

The studies were conducted in various countries within Africa, namely Angola, Cameroon, Congo and Mozambique with one study each [25, 32, 33, 44]. Burkina Faso, Gambia, Ghana and Tanzania contributed two studies each [24, 28, 31, 39–41, 53, 55]. Kenya and Uganda contributed three studies each [19, 26, 42, 43, 54, 63] and Ethiopia had 14 studies [16, 20, 23, 29, 34–38, 59, 65–67, 69]. Most of the studies were conducted in Nigeria with a total number of 19 studies [15, 18, 27, 45–52, 56–58, 60–62, 64, 68]. The majority of the study respondents were parents of children who were within the childhood immunisation schedule range. The studies focused on factors that influence or determine childhood immunisation uptake. The majority of these studies were carried out mainly in West Africa (Nigeria) due to poor childhood immunisation uptake in the nation. This demonstrated that most of the researches were still attempting to determine the main factors that play a role in influencing childhood immunisation uptake, which would enable researchers to address the issues for the development of future health education intervention programs.

### Study type and respondents

The majority of the studies were a cross-sectional study design with a total number of 42 studies, 3 qualitative study design [38, 50, 64], 4 mixed method [23, 37, 41, 68] and 2 case-control [34, 59]. The number of study respondents varied across the studies with 14 being the smallest and 7815 the largest (Table 3).

### Factors associated with immunisation coverage

#### Modifiable factors

The factors that are considered modifiable in this review are obstetric factors, maternal knowledge, maternal outcome expectation, maternal attitude, maternal self-efficacy and environmental factors. These factors were found to statistically influence childhood immunisation uptake in Africa and reported a wide range of AOR and 95% confidence intervals (upper limit and lower limit) from different studies.

In respect to obstetric factors, place of delivery was evident in five studies which are all statistically significant with AOR  2.11–3.13 and 95% confidence intervals (CI): 1.09, 7.13) [26, 44, 63, 65, 67] and three studies also reported postnatal care follow-up with AOR 1.8–5.8; 95% CI: 1.21, 3.16 [20, 59, 63]. Six studies revealed antenatal care follow-up with AOR  2.4–3.7; 95% CI: 1.1, 10.0 [16, 19, 65–67, 69], likewise maternal tetanus toxoid was reported in two studies with AOR  2.43–3.2; 95% CI: 1.10, 10.00 [27, 36].

Many studies reported factors relating to maternal knowledge, for example, knowledge on child immunisation (AOR = 3.3; 95% CI: 1.87, 7.43) [34, 67], knowledge on preventive objective of immunisation (*p* < 0.05) [31], lack of knowledge on vaccines which accounted for 50% reason for non-compliance [57], knowledge on child vaccination schedule, which was reported in three studies with AOR  2.9–4.3; 95% CI: 1.42, 8.0 [26, 35, 59]. Likewise, awareness on immunisation and immunisation programs were also revealed in three studies with AOR  = 1.9–2.8; 95% CI: 1.44, 2.49 [20, 25, 37]. Two studies reported the impact of knowledge of VPDs (AOR  = 2.5; 95% CI: 1.5, 4.2) [16, 61]. For maternal attitude and self-efficacy, three studies reported availability of child immunisation records with AOR  = 2.38–7.7; 95% CI: 1.43, 10. 06) [20, 31, 47]. Likewise, four studies documented fear of vaccine side effects (AOR  = 1.92; 95% CI: 1.01, 3.70) [23, 57, 60, 62], negative beliefs towards childhood immunisation uptake [46], good perception of immunisation (AOR  = 2.60; 95% CI: 1.50, 4.51) [49] and confidence towards vaccine safety (*p* < 0.05) [46].

With regards to maternal outcome expectation, three studies reported knowledge on benefit of childhood immunisation uptake (AOR  = 5.51; 95% CI: 1.52, 19.94) [23, 35, 54] and one study documented severity of VPDs (*p* = 0.045) [45]. With regards to environmental factors, two studies reported religious belief (OR  = 1.65: 95% CI: 1.15, 2.36) [44, 64]. Likewise, two studies also attributed cultural belief [56, 60].

#### Non-modifiable factors

In this review, parental socio-demographic factors (maternal age, maternal education, paternal education, maternal marital status, area of residence, wealth index, number of siblings, religion, ethnicity and family income), child socio-demographic factors (gender, age) and environmental factors (distance to health facility, mode of transportation, accessibility of vaccination site, satisfaction with vaccine services, quality of vaccine provider clients relationship and availability of vaccine) are considered as non-modifiable factors. These factors were found to statistically influence childhood immunisation uptake in Africa and reported a wide range of AOR and 95% confidence intervals (upper limit and lower limit) from different studies.

In respect to parental socio-demographic factors, three studies reported maternal age with AOR  = 1.08–9.54; 95% CI: 1.08, 18.09; *p* < 0.05 [23, 36, 38], nine studies reported maternal educational status which were all statistically significant with AOR  = 3.55–7.50; 95% CI: 1.02, 10.60 [15, 18, 26, 55, 65–67, 69], three studies reported paternal education (AOR  = 3.1; 95% CI: 1.3, 7.4) [16, 48, 59], three studies reported maternal occupation and mothers’ marital status respectively (AOR  = 1.62–3.01; 95% CI: 1.0, 5.8) [16, 24, 58, 65]. Another two studies reported area of residence (AOR  = 2.70; 95% CI: 1.52, 4.81) [18, 26], wealth index was documented in three studies (AOR  = 1.4–2.38; 95% CI: 1.06, 3.73) [20, 65, 69], three studies reported family income (AOR  = 3.2; 95% CI: 1.4, 7.4) [16, 31, 59], one study reported number of siblings (APR  = 1.42; 95% CI: 1.05, 1.71) [25], two studies reported religion (*p* < 0.05) [19, 44], three studies documented nomadic lifestyle (OR  = 11.06; 95% CI: 4.29, 28.54) [26, 43, 68] and ethnicity was also reported in two studies (AOR  = 2.47; 95% CI: 1.28, 4.76) [26, 51]. Regarding child socio-demographic factors, child age was reported in two studies (*p* < 0.05) [25, 56] whilst one study reported gender (OR  = 2.98; 95% CI: 1.21, 7.35) [46]. In relation to environmental factors, distance to health facility was attributed in four studies which were all statistically significant with AOR  = 2.11–3.10; 95% CI: 1.46, 6.30 [16, 26, 44, 67], one study reported mode of transportation (AOR  = 1.54; 95% CI: 1.20, 1.97) [40], accessibility of vaccination site was also documented in one study (AOR  = 4.54; 95% CI: 2.34, 8.77) [29], four studies reported satisfaction with vaccine services (AOR  = 2.63; 95% CI: 1.1, 6.3) [28, 56, 60, 64], quality of vaccine provider clients relationship was recorded in one study (AOR  = 1.86; 95% CI: 1.03, 3.50) [28] and vaccine availability in three studies [56, 60, 68].

## Discussion

The main reason for conducting this systematic review was to identify the factors that influence under-five childhood immunisation uptake among parents. In this review, many factors have been found to influence childhood immunisation uptake and are categorised and reviewed below.

### Parental socio-demographic factors

Maternal age was revealed to be a factor influencing childhood immunisation uptake in a case-control study conducted in Ethiopia that involved 548 children aged between 12 to 23 months, in which mothers over 19 years of age were approximately 10 times more likely to have their children fully immunised compared to mothers under 19 years of age [23]. This may be due to knowledge gained over time on the importance of immunisation by mothers over 19 years of age, combined with the negative impact on children due to lack of immunisation. This finding is supported by two studies conducted in Ethiopia [36, 37].

In this review, maternal education was the most common reported parental socio-demographic factor found to influence childhood immunisation. Mothers with at least a primary or secondary school education were found to be approximately eight times more likely to have their children fully immunised compared to mothers with no formal education [56]. This is more likely due to the fact that as the educational status of mothers improves, the seeking behaviour of children may perhaps increase, which in turn have positive impacts towards childhood immunisation uptake [56]. Furthermore, this could also be a result of changes that accompany maternal education, such as changes in attitudes, traditions and beliefs, increased autonomy and control over household resources which enhance healthcare seeking [26]. Similar findings have been reported in many studies [15, 18, 26, 55, 65–67, 69].

From a cross-sectional study conducted in the Sinana District of Ethiopia, consisting of 591 children aged 12–23 months, paternal education was also found to be statistically associated with the child immunisation status. Children with fathers who had a secondary or higher educational level, were three times more likely to be fully vaccinated compared with children whose fathers had no formal education [16, 48, 59]. As the educational status of fathers improves, the seeking behaviour of children may perhaps increase, which in turn may have positive impacts towards childhood immunisation uptake [48].

Results from a cross-sectional study conducted in the Sinana District of Ethiopia, consisting of 591 children aged 12–23 months, has found maternal occupation to be statistically associated with child immunisation uptake. The proportion of children who were not fully vaccinated was found to be higher among mothers who were housewives [16]. Mothers whose occupation was in the farming sector, were less likely to be totally dependent on their spouses in terms of financial support and were found to be almost twice as likely to complete the immunisation of their children, compared with mothers who were housewives. A likely reason for this would be that mothers would not be reliant on their spouses to provide transport fees in order to take their children to be immunised. They most probably have been exposed to information on the benefit of childhood immunisation uptake through access to media [16]. This finding is supported by another cross-sectional study conducted in Ethiopia [65].

The marital status of a mother was also reported to have an influence towards childhood immunisation. In a descriptive cross-sectional study conducted in Ghana involving 280 mothers, it was found that divorced mothers were 3 times less likely to complete immunisation schedules of their children compared to mothers who were married [24]. In a cross-sectional study conducted in Nigeria involving 232 mothers (children aged 12–23 months), married women were observed to have a significantly adequate knowledge of immunisation which may increase the likelihood of achieving a higher rate of immunised children compared with their counterparts who were either single/divorced/widowed or separated [58]. The marital status of a mother may enhance her knowledge in the sense that those that are married may have more access to education compared to single mothers who may have other responsibilities and would instead tend to put their education aside in order to meet the needs of their children [24]. The supportive role of their partners may also enhance her knowledge if both partners jointly try to find ways to better the health status of their offspring [58].

A cross-sectional study from this review that was conducted in Kenya, which consisted of 298 mothers with children aged 12–23 months, demonstrated that area of residence statistically influenced childhood immunisation uptake. Children who lived in urban areas were 12 times more like to be vaccinated compared to children living in rural communities [26]. In Nigeria, results obtained using NDHS data involving 5754 children aged between 12 and 23 months, also revealed a statistical association between area of residence and child immunisation status. Children residing in urban areas were more likely to be vaccinated compared to children living in rural communities. This may probably be attributed to the fact that parents living in urban areas were more likely to be educated which may increase their knowledge towards the benefit of childhood immunisation uptake compared to parents living in rural areas [18].

Three different cross-sectional studies conducted in Ethiopia among children aged 12–23 months found wealth index to be a factor influencing childhood immunisation uptake. Children born to mothers from a rich index group were found to be twice as likely to be fully vaccinated compared with children from mothers from a poor wealth index group [20, 65, 69]. Rich parents may have more access to media and probably have more exposure to information on the benefit of childhood immunisation uptake.

Family income was found to be another factor influencing childhood immunisation uptake in a cross-sectional study conducted in Burkina Faso consisting of 591 children aged between 12 and 23 months. The study found that if the income of a family is greater than 1000 ETB or 52 USD, it increases the tendency of having children fully vaccinated in that family by approximately three times when compared to a poor family with a lesser income [16]. The family with a higher income may have easier access to Immunisation Centres due to accessible effective transportation options and would have less financial challenges when compared with families with a lower income. This finding was also supported by two cross-sectional study designs [31, 59].

The number of siblings was also found to be a factor affecting childhood immunisation uptake in a cross-sectional study conducted in Angola involving 1209 children under 5 years of age. A family comprising 2 to 3 siblings were more likely to vaccinate their children compared with a family with less than 2 siblings [25]. This may be due to experience gained over time on the importance of immunisation as well as the medical complications that have occurred in children due to lack of immunisation.

Religion has been revealed by studies to be a factor influencing childhood immunisation uptake. From a cross-sectional study conducted in Uganda using Uganda Demographic Health Survey data, childhood immunisation uptake was affected by religious affiliations. Children from Muslim families had a lesser chance of been fully vaccinated compared with children from Catholic families [19]. Likewise, in Mozambique parents with no religious affiliation were found to be twice as likely not to complete their childhood immunisation uptake [44]. This could be due to the circulation of false information obtained via religious networks. This false information may be linked to negative beliefs of vaccines, for example, the belief that vaccines are composed of anti-fertility drugs [70].

Nomadic lifestyle was found to be associated with child immunisation uptake in Kenya. Children born to a family who practice a nomadic lifestyle, were found to be 11 times more likely not to be fully vaccinated compared with children born to a family that do not practice a nomadic lifestyle [26, 43, 68]. The family who practices a nomadic lifestyle may constantly change their location, switching from one place to another where immunisation services may not be readily accessible [26].

Ethnicity was found to be a factor affecting childhood immunisation uptake in Nigeria in which children belonging to the Igbo ethnic group were about three times more likely to be fully vaccinated compared to children belonging to an ethnic group such as Hausa, Yoruba and others [51, 56]. These disparities could be attributed to the factors prevalent at community level, for example, in the Hausa community there is low level of education, high poverty, poor utilisation of antenatal care and home delivery and all these factors are associated with poor immunisation uptake. It could also be due to a misconception regarding the safety of vaccines and fear of vaccine side effects [51]. These findings are consistent with the findings from a systematic review conducted to determine the significant factors of adherence among parents or caregivers of under-five childhood immunisation schedules, even though the review did not specifically focus on Africa [71].

### Child socio-demographic factors

Child gender was found to be associated with child immunisation uptake in Ibadan, Nigeria where a male child is about three times more likely to be immunised compared with a female child [46]. This may be attributed to the beliefs of parents that immunisation will have negative impacts on their daughters when they reach child bearing age.

A contradictory finding was revealed with regards to influence of child age on immunisation uptake. In Nigeria, higher immunisation uptake was observed in children above 1 year of age (84.4%) compared with children below 1 year (63.6%) and this could be due to the fact that some mothers delay child immunisation until their children reach a certain age, due to negative beliefs that their children are too young to be immunised [56]. In Angola, higher immunisation uptake was seen in children who were 1 year of age or less compared with children that were above 1 year of age. This could be attributed to the lectures received by mothers towards benefits of timely childhood immunisation uptake during their antenatal and postnatal care. It is likely that the content of health education delivered to the mothers has changed with more emphasis placed on the benefit of childhood immunisation uptake [25].

### Obstetric factors

Antenatal care follow-up (ANC) was found to be a factor influencing childhood immunisation uptake in a cross-sectional study conducted in Ethiopia that involved 591 children aged between 12 and 23 months. The study showed mothers who frequently attend ANC during their pregnancy were about four times more likely to have their children fully vaccinated compared with mothers who did not attend ANC regularly [16]. This finding is supported by the findings of a study conducted in Uganda and four more studies from Ethiopia [19, 65–67, 69]. Mothers who frequented health facilities during pregnancy may have received counselling on childhood immunisation where the importance of timely childhood immunisation uptake may be prioritised regularly [16].

The postnatal check-up was also found to be an influencing factor towards childhood uptake where children who received a check-up within 2 months after birth were twice more likely to be fully vaccinated compared to those who did not receive a check-up after delivery [20, 59, 63]. This can be attributed to educational sessions that mothers were exposed to during postnatal visits where the importance of timely immunisation of the baby may be emphasised [20].

The maternal tetanus toxoid (MTT) vaccine was also noted to be a factor influencing childhood immunisation uptake. Mothers who received at least one dose of the MTT vaccine were three times more likely to have their children fully immunised compared with mothers who did not receive any dose of the MTT vaccine [27, 36]. This could be attributed to the knowledge that mothers may have obtained regarding the benefit of childhood immunisation uptake during the MTT vaccination in their health centre [27].

Children who were delivered in hospitals were more likely to have a complete vaccination status compared with children delivered at home [26, 44, 63]. Mothers who had hospital deliveries may receive advice after delivery where the importance of timely immunisation of the baby may be emphasised and therefore, they are more likely to receive their vaccines [44].

### Maternal knowledge

Having a good maternal knowledge on child immunisation was revealed to be a predictor for childhood immunisation uptake in a case-control study conducted in Northern Ethiopia. Children of mothers with a good knowledge on childhood immunisation were found to be three times more likely to be completely immunised compared with children whose mothers had a poor knowledge on childhood immunisation [34]. This finding is consistent with the previous systematic review conducted in Sub-Saharan Africa [72].

In Burkina Faso, mothers with a knowledge on preventive objectives of immunisation were more likely to have their children immunised compared with mothers with limited knowledge [31]. In a household-level cluster survey consisting of 7815 children conducted in Nigeria that involved 40 polio high-risk districts of Nigeria, lack of maternal knowledge regarding vaccines was found to be the main reason contributing to poor childhood immunisation uptake which accounted for 50% of reasons for non-vaccination [57].

Maternal knowledge on vaccines and VPDs were also shown to influence childhood immunisation uptake in Southeast Ethiopia, where children whose mothers had a good knowledge on vaccines and VPDs were found to be three times more likely to be fully vaccinated compared with children of mothers who had a poor knowledge of vaccines and VPDs [16]. A similar finding was also found in Nigeria [61]. Maternal knowledge on child vaccine schedules was revealed to statistically influence child immunisation uptake where mothers who had knowledge on schedules of vaccines were found to be four times more likely to fully immunise their children compared with mothers who had no knowledge of vaccine schedules [26, 35, 59]. Mothers with knowledge of immunisation schedules may know the exact time for each childhood immunisation uptake and they might also know the benefit of timely immunisation uptake for their children. Parents who were aware of immunisation and immunisation programs were three times more likely to have their children immunised compared with their counterparts [20, 25, 37]. Childhood immunisation uptake can be negatively affected by the level of knowledge of mothers. The more knowledge they acquire, the higher the tendency of increasing their confidence towards childhood immunisation uptake.

### Maternal attitude and self-efficacy

Studies have shown that mothers who have their child immunisation records were more likely to have their children fully immunised compared with mothers without child immunisation records [20, 31, 47]. In Nigeria, the poor attitude of mothers accounted for 16% of the reasons for poor childhood immunisation uptake [57]. Among 248 defaulting mothers in Ibadan, Nigeria, more than half of this group reported that the reason for defaulting was that they considered childhood immunisation to be a waste of time [46]. The children whose mothers had a positive perception towards vaccine side effects, were twice more likely to be fully immunised compared with children whose mothers had a negative perception towards vaccine side effects [23]. Many studies also reported fear of vaccine side effects influenced immunisation uptake [57, 60, 62]. Mothers who lacked confidence in vaccine safety, were less likely to have their children immunised [46]. Mothers with a good perception on immunisation were three times more likely to have their children fully immunised compared with mothers with a poor perception on immunisation [49]. The attitudes of mothers towards childhood immunisation uptake is influenced by their perception. This in turn can decrease their confidence regarding childhood immunisation uptake.

### Maternal outcome expectations

Mothers who knew the benefits of childhood immunisation were six times more likely to have their children fully immunised compared with their counterparts [23, 35]. Having an expectation towards the protection that follows childhood immunisation significantly influences childhood immunisation [54]. Furthermore, knowing the seriousness of VPDs was also found to be a predictor for non-compliance [45]. The more knowledge mothers acquired with regards to the benefit of child immunisation and consequences of not immunising a child, the higher the tendency of increasing their practice confidence towards childhood immunisation uptake. This finding is consistent with the findings from a systematic review previously conducted that aimed to identify factors associated with immunisation coverage in poor urban areas and slums [73].

### Environmental factors

Environmental factors are classified as social factors and health care systems or logistic factors. Religious belief was revealed to be one of the social factors influencing childhood immunisation uptake in Mozambique. Mothers who considered immunisation as unacceptable in their religion were less likely to have their children fully immunised compared with mothers who did not consider immunisation as unacceptable in their religion [44]. Lack of adequate involvement by religious and traditional leaders in immunisation activities was found to be a reason for immunisation failure in Borno State, Nigeria [64].

In Nigeria, cultural beliefs against immunisation are found to be destructive towards childhood immunisation uptake [56, 60]. This could probably be due to the circulation of false information via the use of either family or religious networks towards vaccines. For example, beliefs that vaccines were composed of anti-fertility drugs and therefore could destroy the eggs of females and cause damage to the reproductive system [70]. Traditional and religious leaders are highly respected and are generally regarded and accepted as the custodians of traditions entrusted to them to provide traditional guidance to their respective communities. Therefore, their involvement in immunisation activities will help increase immunisation acceptance and uptake since the community trust their views on various matters [64].

In Kenya, distance to health facilities was found to affect childhood immunisation uptake among 298 respondents. Children belonging to mothers or caregivers who travelled a short distance to the health facility for immunisation were 18 times more likely to be fully vaccinated compared with children whose mothers or caregivers travelled further to a health facility for their children’s immunisations [26]. This was supported by three studies conducted in Mozambique and Ethiopia [16, 44, 67]. These findings were in line with the previous systematic review conducted in Sub-Saharan Africa [72]. The mode of transportation for immunisation was also found to be an influencing factor where mothers who make use of public transport were twice more likely to have their children fully immunised compared to mothers who walked [40].

Accessibility of vaccination sites was found to be a predictor for childhood immunisation uptake in Southern Ethiopia. Mothers who considered immunisation sites to be accessible were 5 times more likely to have their children fully immunised compared to mothers who did not consider it accessible [29].

Satisfaction with vaccine services was also found to influence childhood immunisation uptake in Tanzania among 380 mothers of children aged 12–23 months. Mothers who are satisfied with vaccine services were about three times more likely to have their children vaccinated compared with mothers who were unsatisfied with vaccine services [28, 56, 60, 64]. The way vaccine providers behave could either enhance or discourage mothers from taking their children for vaccinations [28].

The quality of the vaccine provider and client relationship was also found to be a predictor for childhood immunisation uptake in Tanzania among 380 participants. Mothers who had a positive perception towards quality of vaccine provider and client relationship were twice more likely to have their children fully immunised compared to mothers who had a negative perception towards quality of vaccine provider and client relationship [28]. This could probably be due to the way vaccine providers behave which may either enhance or discourage mothers from taking their children for vaccinations [28].

In Nigeria, the unavailability of vaccines when required was also found to be another reason for defaulting on childhood immunisation uptake [56, 60, 68]. Mothers may have spent a considerable amount of money in order to access health care on several occasions. However, the service was not always available which resulted in them becoming discouraged and they failed to complete the immunisation uptake of their children [56].

The findings of the study will benefit policy makers in making decisions and formulating appropriate guidelines and policies with regards to childhood immunisation uptake in Africa. These findings can also enable other researchers to plan an appropriate health education intervention encompassing an appropriate health behavioural theory to address the factors affecting childhood immunisation uptake among African countries. The strengthening of childhood immunisation policies, strategies and further research examining the causal relationship on childhood immunisation in Africa are required.

Social cognitive theory (SCT) is one of the health theories commonly used in health education interventions [74, 75] which describes human behaviour through the influence of personal and environmental factors [76]. The theory accounts for human behaviour, cognition and environment and is the only health theory that takes into account reciprocal interaction, unlike other theories such as Information-Motivation-Behavioural Skills model [74, 77]. In this systematic review, there are many factors that has been found to influence childhood immunisation uptake in Africa such as personal factors (parental sociodemographic factors, obstetric factors, knowledge gaps, negative attitudes, outcome expectation and lack of self-efficacy) and environmental factors (social factors and healthcare system) [16, 18–20, 23–29, 31–64, 70]. Therefore, using this theory may enable researchers to address both the personal and environmental factors influencing childhood immunisation uptake.

The majority of our included studies achieved at least high scores [16, 18, 20, 23–29, 31–38, 40, 42–45, 48, 50–52, 55, 57–59, 61–69] and the overall risk of bias was moderate in ten studies [15, 19, 39, 46, 47, 49, 53, 54, 56, 60]. Moreover, none of the included studies revealed either a serious or low risk of bias.

Our study has acknowledged some limitations. This review may be prone to publication bias as grey literature/unpublished literature and studies that are not published in the English language were not considered. Conducting a quantitative meta-analysis in this systematic review may be very important for analysing quantitative trends. The majority of the literature cited in this review is observational in nature and therefore, this study is lacking evidence for causation. Furthermore, many studies relied on survey data which may increase the risk of nonresponse bias or selection bias.

## Conclusion

In conclusion, various factors influencing childhood immunisation uptake in Africa were identified from this systematic review. The factors were categorised into two main groups, modifiable and non-modifiable factors which were later divided further. The modifiable factors (obstetric history, maternal knowledge, maternal attitude and self-efficacy and maternal outcome expectation) were revealed as having a direct relationship with the childhood immunisation uptake. Many factors and results attained from this review could enable the researchers to further understand and develop necessary intervention in order to address the issue of the factors influencing childhood immunisation uptake. Finally, we recommend an immunisation health education intervention among pregnant women focusing on the significant findings from this systematic review which may hopefully improve childhood immunisation uptake among countries with poor coverage in Africa.

## Supplementary Information

**Additional file 1: Supplemental file 1.** Database search terms used; Fig. 1: PRISMA flow diagram for the identification, screening, eligibility, and inclusion of studies; Table 1: Eligibility criteria table; Table 2: Operational definition; Table 3: Summary of systematic review factors influencing childhood immunisation uptake.

## Data Availability

All data generated during this study are included in this article.

## Abbreviations

AOR: Adjusted Odd Ratio
OR: Odd Ratio
CI: Confidence Interval
APR: Adjusted Prevalence Rate
EDHS: Ethiopia Demographic Health Surveillance
NDHS: Nigeria Demographic Health Surveillance
FHDSS: Farafenni Health and Demographic Surveillance
NUHDSS: Nairobi Urban Health and Demographic Surveillance System
THDSS: Tanzania Health and Demographic Surveillance System
NHDSS: Nouna Health and Demographic Surveillance System
UDHSS: Uganda Demographic Health Surveillance System
TT: Tetanus Toxoid
ANC: Antenatal Care
PNC: Postnatal Care
VPDs: Vaccine Preventable Diseases
EPI: Expanded Program on Immunization
NPI: National Program on Immunization
RI: Routine Immunization
HEWs: Health Extension Workers
OPV: Oral Polio Vaccine
EPI: Expanded Programme on Immunization
APR: Adjusted Prevalence Ratio
