# Supplementary material for: Factors influencing childhood immunisation uptake in Africa: a systematic review
Source: BMC Public Health. 2021 Jul 28;21:1475. doi: 10.1186/s12889-021-11466-5 (PMC8320032; doi:10.1186/s12889-021-11466-5)
Supplement: Supplementary file 1 — Additional file 1: Supplemental file 1. Database search terms used; Fig. 1: PRISMA flow diagram for the identification, screening, eligibility, and inclusion of studies; Table 1: Eligibility criteria table; Table 2: Operational definition; Table 3: Summary of systematic review factors influencing childhood immunisation uptake. [file 12889_2021_11466_MOESM1_ESM.docx]

1. “Childhood immunization uptake”
2. factors
3. influencing
4. affecting
5. child
6. new born
7. infant
8. baby
9. immunization
10. vaccines
11. vaccination
12. Pentavalent vaccine
13. Penta vaccine
14. Bacillus Calmette Guerin vaccine
15. BCG
16. Diphtheria Tetanus and Pertussis
17. DTP
18. Oral polio vaccine
19. OPV
20. Measles vaccine
21. Yellow fever vaccine
22. Pneumococcal Conjugate vaccine
23. PCV
24. Hepatitis B vaccine
25. Hep B vaccine
26. Uptake
27. Adherence
28. Compliance
29. Coverage.
30. 3 OR 4
31. 5 OR 6 OR 7 OR 8
32. 9 OR 10 OR 11 OR 12 OR 13 OR 14 OR 15 OR 16 OR 17 OR 18 OR 19 OR 20 OR 21 OR 22 OR 23 OR 24 OR 25
33. 26 OR 27 OR 28 OR 29
34. 1 AND 30 AND 31 AND 32 AND 33
